# Validity and Reliability of the Treatment Adherence Questionnaire for Patients with Hypertension

**DOI:** 10.17533/udea.iee.v37n3e09

**Published:** 2019-10-23

**Authors:** Natalia Esquivel Garzón, Luz Patricia Díaz Heredia

**Affiliations:** 1 Nurse, PhD candidate. Universidad Nacional de Colombia, Bogotá, Colombia. Email: nesquivelg@unal.edu.co Universidad Nacional de Colombia Universidad Nacional de Colombia Bogotá Colombia nesquivelg@unal.edu.co; 2 Nurse, PhD. Associate Professor, Universidad Nacional de Colombia, Bogotá, Colombia. Email: lpdiazh@unal.edu.co Universidad Nacional de Colombia Universidad Nacional de Colombia Bogotá Colombia lpdiazh@unal.edu.co

**Keywords:** essential hypertension, treatment adherence and compliance, surveys and questionnaires, psychometrics, validation studies., esencial, cooperação e adesão ao tratamento, inquéritos e questionário, psicometria, estudos de validação.

## Abstract

**Objective.:**

To determine the validity and reliability of the Treatment Adherence Questionnaire for Patients with Hypertension (TAQPH), Spanish version, designed by Chunhua Ma *et al*.

**Methods.:**

This study was carried out in the city of Ibagué (Colombia) and the test validation determined validity (face, content, and construct) and reliability. Face and content validity were conducted through expert judgment, using Fleiss’ Kappa Coefficient statistical tests and modified Lawshe’s content validity index. The construct validity and the reliability test had the participation of 220 people with diagnosis of primary hypertension. Reliability was calculated through Cronbach’s alpha statistical test.

**Results.:**

In the face validity, the instrument reported a Fleiss’ Kappa index was 0.68 in comprehension, 0.76 in clarity, and 0.64 in accuracy, interpreted as a substantial agreement. The content validity index was satisfactory with 0.91; el exploratory factor analysis reported six factors with a total variance explained of 54%. Cronbach’s alpha for the total scale was 0.74.

**Conclusion.:**

The Spanish version of the TAQPH is a valid and reliable scale to evaluate adherence to treatment in patients with primary hypertension.

## Resumo

**Objetivo:**

Determinar a validez e confiabilidade do questionário para medir a aderência ao tratamento em pacientes com hipertensão arterial (*Treatment Adherence Questionnaire for Patients with Hypertension -* TAQPH), versão em espanhol, desenhado por Chunhua Ma *et al.*

**Métodos.:**

Estudo de validação de provas que se realizou na cidade de Ibagué (Colômbia) no qual se determinou a validez (facial, de conteúdo e de construto) e a confiabilidade. A validez facial e de conteúdo se efetuou mediante o juízo de especialistas, utilizando as provas estatísticas de Coeficiente Kappa de Fleiss e índice de Lawshe modificado. Na validez de construto e prova de confiabilidade participaram 220 pessoas com diagnóstico de hipertensão arterial. A confiabilidade se calculou mediante a prova estatística de alfa de Cronbach.

**Resultados.:**

Na validez facial, o instrumento reportou um índice de Kappa de Fleiss de 0.68 em compreensão, 0.76 em claridade e 0.64 em precisão, interpretado como um acordo substancial. O índice de validez de conteúdo foi satisfatório com 0.91, a análise fatorial exploratório reportou seis fatores com uma variação total explicada de 54%. O alfa de Cronbach foi de 0.74 para a escala total.

**Conclusão.:**

A versão em espanhol de TAQPH é uma escala válida e confiável para a avaliação da aderência ao tratamento em pacientes com hipertensão arterial.

## Introduction

Among the group of chronic noncommunicable diseases characterized for being long lasting and of slow progression, we find cardiovascular diseases, attributable to risk factors among which there is primary hypertension.(1) Together, cardiovascular diseases represent the first cause of morbidity and mortality in the world and affect principally low- and medium-income countries,(2) causing disability and premature death, which leads to increased disease burden due to this group of diseases.

One of the indicators of success in the treatment of primary hypertension is adherence,(3) in spite of this, it is estimated that between 50% and 80% of the patients who receive antihypertensive drug treatment have low adherence,(4) approximately 10% omits a dosage of medication on a given day and nearly half of those diagnosed suspend the medication during the first year of treatment.(5) This situation can lead to inadequate control of the blood pressure, which in the future could cause cardiovascular complications, hospital readmissions, premature deaths due to disability, besides the costs implied for care in the health system.(6) Lack of adherence may take place when starting a new prescription, in the implementation of the treatment, or during the persistence and it is manifested by the difficulty to start the treatment, abandonment or incomplete compliance, non-attendance to consultations and interconsultations, lack of modification of lifestyles, and/or inadequate follow up of the recommendations, a situation that affects the course of the disease.(7)^.^

To establish objectively the concordance between the therapeutic orientations and their execution by the individual, it is necessary to measure the degree of adherence to treatment; for such, different methods have been proposed, which are classified into direct and indirect, without any of them considered the gold standard.(8) Among the indirect methods, there are the questionnaires, like: the Medication Adherence Scale by Morisky *et al.,*(9) the Brief Medication Questionnaire,(10) the Hill-Bone Compliance to High Blood Pressure Therapy Scale,(11) the Martín-Bayarré-Grau Questionnaire,(12) the Maastricht Utrecht Adherence in Hypertension (MUAH),(13) the evaluation of the “Therapeutic Behavior: disease or lesion”, which corresponds to a standardized classification of the patient’s results established by the North American Nursing Diagnosis Association (NANDA), and the Compliance Scale of Hypertensive Patients;(14) however, some of these instruments measure exclusively adherence to pharmacological treatment,(9) while others center on facilitators or barriers to adherence, such as beliefs on the effects of the medications,(10) behavior related with sodium intake, compliance with treatment and medical appointments,(11) attitudes and therapeutic coping,(13) autonomy upon the treatment and participation by the patient,(12) lifestyle, attitude toward the disease, and responsibility in the treatment.(14) In spite of the availability of these instruments, none estimates adherence in terms of the pharmacological treatment and lifestyle, fundamental aspects in controlling primary hypertension, besides being included in the definition of adherence proposed the World Health Organization, which considers it as the measure in which the behavior of a person who takes medications, follows a diet and/or undergoes lifestyle changes corresponds with the recommendations agreed with a medical care provider.(15) 

The Treatment Adherence Questionnaire for Patients with Hypertension (TAQPH), designed and validated by Nursing Dr. Chunhua Ma *et al.,*(16) measures adherence to pharmacological and non-pharmacological treatment. This 28-item instrument is originally composed of six factors (F1 - *Medication* = 9 items, F2 - *Diet =* 9 items*, F3 - Stimulants* = 3 items, F4 - *Weight control* = 2 items, F5 - *Exercise* = 2 items, *F6 - Stress relief* = 3 items) and has four Likert-type response options (1 = never, 2 = sometimes, 3 = most of the time, 4 = all the time), the score is the sum of the value obtained in each item, a higher score means better adherence. The evaluation of its factor validity showed that the six factors explain 62.5% of the total variance; besides, having criterion validity when compared with the Medication Adherence Scale by Morisky (*r* = 0.76) and with the General Self-efficacy Scale (*r* = 0.69) and high reliability (0.86). This questionnaire does not have a Spanish version and, given that a valid and reliable instrument was required adapted to the context in which it will be used, which is Colombia, this study was conducted to determine the validity and reliability of the TAQPH.

## Methods

This study was conducted between March 2017 and April 2018 in the city of Ibagué in Tolima, Colombia. The development phase included the translation, linguistic and cultural adaptation, pilot test, and back-translation. The confirmation phase performed the face, content, and construct validity, along with the internal consistency evaluation. 

*First phase: Translation and cultural adaptation.* A direct translation was carried out from English to Spanish by two official translators and a Colombian bilingual nurse (Spanish-English), different from the principal researcher with knowledge on the theme of adherence. This procedure focused on adaptation of meanings more than a textual or literal translation. Thereafter, the combination of translations was conducted along with the synthesis of a first version revised by a committee comprised of the principal researcher, a nurse with a Masters degree in cardiovascular care, and a linguist experienced in the production of written texts and knowledge of medical terminology, who were in charge of judging the semantic, idiomatic, conceptual and cultural equivalence. From the aforementioned, a second preliminary version was generated, which was applied to a group of 10 adult volunteers with different educational levels, to collect information regarding comprehension of the instructions and items, register the time needed to fill out the instrument and identify possible errors of content or form that were corrected prior to moving on to the following phase. Lastly, the back-translation was conducted by a third official translator after expert approval of the face and content validity; this was subsequently sent to the author for her final approval. 

*Second phase: Face validation.* This was carried out by two groups: one made up of 20 patients, men and women, over 18 years of age, with diagnosis of primary hypertension, registered in a follow-up program for chronic patients, which were not included in the principal study and another made up of panel of four experts, selected for their academic formation with graduate degree or Masters in the health area and clinical experience over five years, who evaluated the instrument by considering three scoring criteria: comprehension, clarity, and accuracy. With the results, the Fleiss’ Kappa index was calculated, which permitted determining agreement among observers correcting randomness. The results were interpreted according to that recommended by Landis and Koch,(17) who consider satisfactory those items obtaining values ranging between 0.61 and 0.80, recognized as substantial agreement.

*Content validity.* The same four experts who participated in the face validity participated in the content validity, evaluating each of the items with the following criteria: “essential”, “useful but not essential”, and “not necessary”. With the data obtained, the Content Validity Ratio (CVR) was calculated, which was defined as the proportion of agreements in the essential category with respect to the total number of evaluators. Additionally, the Content Validity Index (CVI) of the whole instrument was calculated. The aforementioned is supported in the Lawshe model modified by Tristán(18) that establishes that CVR value ≥0.58 is sufficient to consider an item as acceptable, independent of the number of evaluators.

*Construct validity.* To apply the questionnaire, a type of convenience sampling was applied. The Gorsuch(19) proposal of five subjects per item was taken as reference for sample size selection. According to the aforementioned, the study opted for a calculated sample of 216 participants, considering seven people per item plus an additional 10% in case of losses, obtaining in the end the participation of 220 patients. Inclusion criteria were: men and women over 18 years of age, with diagnosis of primary hypertension, registered in a cardiovascular risk program, with more than six months of treatment. The study excluded patients with secondary hypertension and those who, besides having primary hypertension, were diagnosed with diabetes mellitus.

To verify the data fit prior to the exploratory factor analysis, Bartlett's sphericity tests were applied (*p*<0.0001), and the sampling adequacy measure with the Kaiser-Meyer-Olkin (KMO) index. Thereafter, an exploratory factor analysis was performed with the principal components extraction method and the Kaiser-Varimax rotation method; the analysis was forced to six factors in correspondence to those described by the instrument’s author. Taking as reference that defined by Bandalos,(20) the criteria used to determine the amount of factors extracted in the instrument were own values >1 and with loads >0.30. The data were processed by using the SPSS statistical program, version 22.0. Lastly, the internal consistency was determined from Cronbach’s alpha calculation, considering that a Cronbach’s alpha of 0.70 or higher was adequate if the objective of the scale is for use in research.(21)

*Ethical considerations.* Throughout the development of the research process, ethical principles were complied; the study participants signed the informed consent after receiving information about the study objective and accepting voluntarily to participate in the research. Permission was obtained from the instrument’s author to carry out the translation process, cultural adaptation, validity, and reliability from the internal consistency. 

## Results

*Translation and cultural adaptation.* It was found that all the questions of the instrument in English began with the modal verb “would”, which gave for a conditional nature; with accompaniment by the instrument’s author, it was clarified that the instrument sought to establish the behavior in relation with taking medications, monitoring the diet, and changes in lifestyle the person had had until the moment. Due to the aforementioned, it was established that the most adequate verbal tense to use in the Spanish version would be the perfect tense, given that it indicates an action in the past that continues in the present. At the end of this phase, a version was available adapted to the Colombian language and cultural context. 

*Face validity.* In relation with the scale accuracy, the agreement index - measured through Fleiss’ Kappa - reported for the instrument in general substantial agreement, summarized in values of comprehension 0.68, clarity 0.76, and accuracy 0.64. The evaluation made by the 20 individuals reached an agreement percentage of 98%; 96% for clarity; and 95% for accuracy. From these results, corresponding adjustments were performed, incorporating the recommendations made by the experts and the patients in terms of phrasing the items.

*Content validity.* The content validity kept in mind the judgment by experts or the evaluation panel. The results corresponding to CVR indicated that all the items could be acceptable, given that values obtained were >0.58, ranging between 0.75 and 1. The content validity index for the 28 items was of 0.91, a value considered acceptable and, as consequence of these findings, all the items were maintained. 

*Construct validity.* Of the 220 subjects participating in this stage, the majority were women (72.3%); the mean age was 65.1 years, ranging between 50 and 82 years; nearly three out of every four (73.1%) patients had a maximum of secondary education; 42% were dedicated to household work; and 62% were married or in common-law relationships (Table 1).

**Table 1:** Sociodemographic characteristics of the 220 patients with primary hypertension

Characteristics	Values
Age; *mean ± SD, range*	65.1±7.4, 50-82
Sex; *n (%)*	
Female	159 (72.3)
Male	61 (27.7)
Level of schooling; *n (%)*	
Primary	63 (28.6)
Secondary	98 (44.5)
Technical or university	59 (26.8)
Occupation; *n (%)*	
Employed	37 (16.8)
Home	93 (42.3)
Pensioned	52 (23.6)
Independent worker	38 (17.3)
Marital status; *n (%)*	
Married or common-law	138 (62.7)
Single, divorced, or widow(er)	82 (37.3)

Prior to the factor analysis, Bartlett’s sphericity test was conducted, reporting a chi-square value = 2171.3 (*p* <0.001); the Kaiser-Meyer-Olkin sampling adequacy measure was 0.74, which proved the general adequacy of the matrix and indicated that it was possible to perform the factor analysis. The anti-image matrix in the extraction through the principal components method and Varimax rotation showed that 54.5% of the total variance was contained in six factors (F1 = 16.0%, F2 = 31.2%, F3 = 38.0%, F4 = 44.6%, F5 = 50.0%, and F6 = 54.5%), making it satisfactory and sufficient to identify the dimensions that establish relations among the items that conform the instrument. The first factor had 8 items, the second 5 items, the third 6 items, the fourth 2 items, the fifth 3 items, and the sixth 4 items. The exploratory factor analysis showed reorganization of the items that would integrate each of the six components, as noted in Table 2.

**Table 2:** Rotated matrix of principal components of the items of the questionnaire to measure adherence in patients with primary hypertension

Item	F1	F2	F3	F4	F5	F6
1. Have you taken the medications according to the frequency indicated in the formula provided by the physician?	0.680	0.224	0.042	0.011	0.158	0.149
2. Have you taken the medications according to the dosage indicated by the physician?	0.693	0.064	0.136	0.015	0.109	0.071
3. Have you taken the medications according to the schedule indicated by the physician?	0.425	0.379	0.235	0.195	0.159	0.003
4. Have you taken the medications for a long period without interruptions, according to indications provided by the physician?	0.684	0.087	0.107	0.101	0.078	0.032
5. Have you taken the medications according to indications by the physician, without increasing or diminishing the dosage?	0.656	0.113	0.014	0.038	0.074	0.011
6. Have you continued taking the medications even if you don’t have symptoms of hypertension?	0.732	0.092	0.106	0.050	0.208	0.035
7. Have you forgotten to take your medications?	0.254	0.382	0.014	0.001	0.299	0.089
8. Have you suspended the medications when you have felt that symptoms have improved?	0.730	0.051	0.051	0.059	0.048	0.018
9. Have kept using the medications in spite of feeling that the symptoms have worsened?	0.809	0.078	0.019	0.084	0.056	0.006
10. Have you complied with a low-salt diet?	0.063	0.649	0.117	0.181	0.074	0.074
11. Have you complied with a low-fat diet? Reducing consumption of fried preparations, sauces, dressings, sausages (cold cuts - Mortadella - fast foods in general)	0.069	0.745	0.225	0.017	0.072	0.092
12. Have you complied with a low-cholesterol diet? Reducing consumption of red meats, chicken skin, eggs, sauces (mayonnaise - tomato sauce - industrial vinaigrettes), oil, lard and butter).	0.038	0.774	0.234	0.014	0.049	0.091
13. Have you diminished consumption of sugar and sweets?	0.020	0.645	0.134	0.133	0.031	0.216
14. Have you increased consumption of fiber? Such as papaya, pineapple, soursop, peaches, pears, and apples; also cereals, like oats, quinoa and bran.	0.070	0.191	0.661	0.059	0.362	0.046
15. Have you increased consumption of fresh vegetables?	0.011	0.178	0.761	0.019	0.115	0.192
16. Have you increased consumption of fresh fruits?	0.002	0.170	0.786	0.078	0.288	0.123
17. Have you increased consumption of grains? Including beans, chickpeas, lentils, peas. Besides dry nuts, like peanuts and almonds.	0.505	0.381	0.566	0.364	0.332	0.182
18. Have you increased consumption of low-fat dairy products?	0.074	0.252	0.516	0.049	0.231	0.093
19. Have you diminished the consumption of coffee?	0.031	0.257	0.035	0.197	0.412	0.189
20. Have you limited consumption of alcoholic beverages?	0.181	0.119	0.085	0.182	0.649	0.138
21. Have you stopped smoking?	0.077	0.174	0.165	0.115	0.581	0.020
22. Have you performed physical exercise at least five times per week?	0.088	0.143	0.105	0.904	0.125	0.069
23. When performing physical exercise, have you dedicated at least 30 minutes to it?	0.071	0.128	0.069	0.908	0.094	0.072
24. Have you been able to control the amount of food you consume?	0.001	0.166	0.062	0.053	0.045	0.623
25. Have you maintained your body weight under control?	0.018	0.340	0.019	0.067	0.337	0.574
26. Have you set aside daily time for relaxation for yourself?	0.065	0.100	0.316	0.194	0.123	0.635
27. Have you recurred to some forms to relieve stress or tension?	0.126	0.005	0.422	0.385	0.234	0.318
28. Have you controlled yourself emotionally in light of sudden events?	0.031	0.111	0.085	0.228	0.253	0.474

The principal components matrix showed a grouping of the items into six factors, which does not coincide precisely with those defined by the instrument’s author, hence, the proposal to rename each factor with a label according to the conceptual relationship among the items, thus: First: use and follow up of the pharmacological treatment; Second: follow up of diet restrictions; Third: follow up of a healthy diet; Fourth: capacity to perform physical exercise regularly; Fifth: control in the use of stimulant substances, and Sixth: management of stressful situations and control of body weight. 

*Reliability analysis.* The instrument reported a Cronbach’s alpha coefficient of 0.74, which indicates an acceptable level of the questionnaire’s reliability.(22)

## Discussion

This study describes the validity and reliability process, evaluated with the internal consistency of the TAQPH instrument, to measure adherence to pharmacological and non-pharmacological treatment (lifestyle), in patients with primary hypertension. The adapted version had modifications without affecting the instrument’s original structure to maintain the semantic equivalence, a fact validated by its author. Face validity permitted evaluating the clarity, comprehension, and accuracy of each items in the instrument, obtaining substantial agreement among the experts. The content validity obtained an index of 0.91, a value considered adequate, showing the pertinence of each of the items in the conformation of the instrument, according to that defined by Lawshe and then modified by Tristán.(18)

It is highlighted that in the study evaluating psychometric properties by Ma *et al.,*(16) when performing the factor analysis through the principal components method and Varimax rotation, a six-factor solution was obtained, and of the 33 items that initially comprised the instrument; five were eliminated due to having factor loads <0.4, thus, the final version of the instrument is conformed by 28 items. To analyze the items integrating each of the instrument’s factors, the study kept in mind the order and content of the questions of the final version published in English, which was compared with the results from this research. 

Six factors were identified and renamed, thus: Factor 1 - *Use and follow up of the pharmacological treatment*, comprised by eight items (1, 2, 3, 4, 5, 6, 8, and 9); Factor 2 - *Follow up of the diet restrictions*, which grouped five items (7, 10, 11, 12, and 13), representing the actions performed by the individual to diminish or avoid consuming foods harmful to health, except for item 7 that corresponds originally to the dimension “medications”; Factor 3 - *Follow up of a healthy diet*, made up of six items (14, 15, 16, 17, 18, and 27) that reflect actions related with the consumption of an adequate diet for people with primary hypertension, except for item 27 that migrated from dimension 6 denominated by Ma *et al.,*(16) as “stress management”; Factor 4 - *Capacity to perform physical exercise regularly,* with items 22 and 23, had the particularity of presenting high correlation coefficients and maintaining the only two items that correspond conceptually and conform the dimension denominated by the author as “exercise”; Factor 5 - *Control in the use of stimulant substances*, grouped three items (19, 20, and 21) that correspond conceptually to the dimension denominated originally “stimulants; and, lastly, Factor 6 - *Management of stressful situations and weight control* was conformed by four items (24, 25, 26, and 28). In the original version, the six factors were related with "medication" (9 items), "diet" (9 items), "exercise" (2 items), "stimulants" (3 items), "weight control" (2 items), and "stress relief" (3 items). As noted, the dimension originally denominated as “diet” was divided in this study into two factors distributed in the second and third factors, which maintain theoretical coherence among the items that comprise them, except for item 7 that is related more with factor 1 “medications” and item 27 related more with factor 6 “stress relief”. 

The study by Dehghan *et al.,*(23) which validated the TAQPH instrument in Persian, obtained a six-factor instrument with a total of 25 items, given that items 4, 19, and 20 were eliminated because they did not report factor loads in any factor. In addition, they introduced the following denominations for each of the factors: “diminish the unsafe diet and weight control" (7 items), "medication" (5 items), "increase the safe diet" (5 items), "stimulation and exercise" (3) items), “avoid self-medication” (3 items), and “recover from stress” (3 items).

In this study, a detailed analysis of the parameters used to evaluate the internal structure of the TAQPH instrument found that the item-factor correlation coefficient was lower than that established by the instrument’s author; however, a value of 0.30 tends to be considered an acceptable minimum.(20) Additionally, to evaluate the coefficients, aspects must be evaluated, like sample size and number of items; in general, with a higher number of subjects, the coefficients can be lower, although these should not be <0.30 to consider them as representative of a factor. With respect to the conformation of Factor 4 - *Capacity to perform physical exercise regularly*, although it had two items, it was maintained as independent factor, given that the underlying concept is the physical activity, which differentiated it from the other factors. 

The total variance explained in this research was lower, considering that the six factors provided 54.51% of the total variance, compared with 62.54% reported in the original study conducted by Ma *et al.,*(16) and with the 60.3% reported by Dehghan *et al*.,.(23) The internal consistency was estimated through Cronbach’s alpha at 0.74, considered adequate,(21) against α = 0.86 reached in the study conducted by the instrument’s author in its English version and the α = 0.80 obtained by Dehghan *et al*.,(23) that may be due to the variants in the cultural context and language.

Among the limitations of the study is that the sample was obtained from a single municipality in urban population and did not include patients from other regions or rural area, which must be kept in mind for the generalization of the results. 

The conclusion, herein, is that the questionnaire to measure treatment adherence in patients with hypertension, in its Spanish version, is a valid and reliable instrument to measure the construct of adherence; the results show the strength of the instrument for its use by health professionals, both in the clinical setting and in research, to identify the degree of adherence and develop intervention strategies aimed at promoting and maintaining the health of individuals who endure this condition. 
